# Comparison of dobutamine and levosimendan for treatment of sepsis-induced cardiac dysfunction: A protocol for systematic review and meta-analysis

**DOI:** 10.1097/MD.0000000000029092

**Published:** 2022-03-18

**Authors:** Jun Guo, Xianhuan Zhang, Yanan Zhu, Qiong Cheng

**Affiliations:** *Department of Emergency, Taizhou Hospital of Zhejiang Province, Zhejiang Province, China.*

**Keywords:** cardiac dysfunction, dobutamine, levosimendan, meta-analysis, sepsis

## Abstract

**Background:**

Levosimendan and dobutamine are extensively used to treat sepsis-induced cardiomyopathy. Previous studies on whether levosimendan is superior to dobutamine are still controversial. We performed a protocol for systematic review and metaanalysis to compare the efficacy and safety of levosimendan versus dobutamine for the treatment of sepsis-induced cardiomyopathy.

**Methods:**

This protocol follows the Preferred Reporting Items for Systematic Reviews and Meta-Analyses protocol statement. We will search the following databases: PubMed, Embase, Web of Science, Cochrane Library, China National Knowledge Infrastructure, Wanfang Database, Weipu Journal Database, and Chinese Biomedical Literature Database. The search time will be set from database establishment to February 2022. After literature screening, 2 reviewers will extract data from the respects of general information, methodology, and results. Risk of bias is assessed using the Cochrane Risk of Bias Tool for randomized controlled trials. We will apply RevMan 5.4 software for statistical analysis.

**Results:**

The results will be submitted to a peer-reviewed journal once completed.

**Conclusion:**

Septic patients with myocardial dysfunction may partly benefit from levosimendan than dobutamine, mainly embodied in cardiac function improvement.

## 1. Introduction

The latest several decades have witnessed the progress n the treatment of severe sepsis and septic shock, acute organ dysfunction and consequential multiple organ dysfunction, however, cardiovascular dysfunction due to severe infection is still a major contributor to sepsis related morbidity and mortality.^[1,2]^ Cardiovascular dysfunction induced by severe sepsis is characterized by signs of distributive shock and septic cardiomyopathy consisting of bi-ventricular myocardial contractility impairment and diastolic dysfunction. The characteristics of septic cardiomyopathy include left ventricular dilatation, depressed ejection fraction and recovery during 7 to 10 days. In severe sepsis and septic shock, myocardial depression is the manifestation of septic cardiomyopathy and may attribute to the overwhelming production of inflammatory cytokines, mitochondrial dysfunction, and decreased myofibrillar sensitivity to calcium.^[3,4]^

International sepsis guidelines have been adopted worldwide, and it is widely accepted that the standard treatment for sepsis should concentrate on infection control and optimization of hemodynamic parameters by fluid resuscitation and vasopressor therapy including noradrenaline and vasopressin.^[5]^ These standard treatment also apply to septic cardiomyopathy. In addition, using dobutamine to increase the cardiac index is recommended by international sepsis guidelines. However, several studies have demonstrated that the use of dobutamine to increase cardiac output did not improve microcirculation, peripheral perfusion, or the outcome of septic shock patients,^[6]^ and even increased the 90-day mortality rate.^[7]^

Another inotropic agent is levosimendan, a Ca^2+^ sensitizer and inodilator, which has been used successfully in the management of acute heart failure.^[8]^ Levosimendan not only has inotropic and vasodilator effects, but also has anti-inflammatory and antiapoptotic effects.^[9,10]^ Whether levosimendan is superior to dobutamine remains a highly contentious issue. Therefore, we performed a protocol for systematic review and meta-analysis to compare the efficacy and safety of levosimendan versus dobutamine for the treatment of sepsis-induced cardiomyopathy.

## 2. Methods

### 2.1. Study registration

The study procedure of this protocol is followed the Preferred Reporting Items for Systematic Review and Meta-analysis Protocols.^[11]^ We have registered at the OSF with registration number 10.17605/OSF.IO/PKWTB. Since this study is on the basis of published studies, ethical approval is not required.

### 2.2. Search strategy

We will search the following databases: PubMed, Embase, Web of Science, Cochrane Library, China National Knowledge Infrastructure, Wanfang Database, Weipu Journal Database, and Chinese Biomedical Literature Database. The search time will be set from database establishment to February, 2022. Ongoing or unpublished studies which have been registered will be considered. The search strategies are developed by 2 of our team members and further decided after the discussion of all team members. The following Medical Subject Headings (MeSH) terms and free text are used: “levosimendan,” “dobutamine,” “sepsis,” and “cardiac dysfunction.” Additionally, to avoid missing relevant studies, the references of the included studies will also be searched via Google Scholar. All the search results will be imported into EndNote V.X9, Clarivate Analytics, USA.

### 2.3. Inclusion and exclusion criteria

Randomized controlled trials with the following criteria were included:

1. the study population was adult individuals with sepsis,
2. the study compared levosimendan with dobutamine, and
3. outcome: the change (before-after comparison to the baseline) of cardiac function parameters at the time point of 24-hours, including cardiac index, left ventricular ejection fractions and left ventricular stroke work index.

The exclusion criteria for this study were as follows:

1. conference abstracts, case reports, comments, editorials, etc.;
2. for multiple publications that have been determined to be reported in the same clinical study, the publication with the most complete publication data is qualified; and
3. the literature information was insufficient to extract the required useful data.

### 2.4. Data extraction

The following data were extracted for each article:

1. bibliographical data, including authors and year of publication;
2. clinical trial features such as sample size, study flow, recruitment method, criteria for inclusion and exclusion, primary measures, time and point of assessments, and duration of the intervention;
3. participant characteristics such as age, sex, and so on;
4. patient background, including country and race; and
5. study drop-out rate and handling of missing data.

### 2.5. Risk of bias assessment

Two investigators will separately assess the risk of bias of the included studies using the Cochrane risk of bias assessment tool.^[12]^ The evaluation of each study mainly included the following 7 aspects: random sequence generation, allocation hiding, blinding of participants and personnel, blinding of outcome assessment, incomplete outcome data, incomplete outcome data, selective outcome reporting, and other biases. Finally, the bias of the study will be rated on 3 levels: “low,” “high,” and “ambiguous.” In the event of disagreement, a consensus will be reached following a discussion by the reviewers. A third reviewer will be available to arbitrate the controversies that remain unresolved.

### 2.6. Statistical analysis

In this study, we will apply RevMan 5.4 software for statistical analysis. The risk ratio and 95% confidence intervals were collected for enumeration data, while the mean difference or standardized mean difference and 95% confidence interval were used to calculate continuous outcome data. The heterogeneity of the data was tested by calculating *I*^2^ statistics. The study was not considered to have a large heterogeneity when the *I*^2^ value was less than 50%. When the *I*^2^ value exceeded 50%, there was significant statistical heterogeneity among the trials. When there is homogeneity in the merged outcome results across sufficient studies, a meta-analysis will be conducted. Otherwise, we performed a subgroup analysis to explore the causes of the heterogeneity.

## 3. Discussion

Septic cardiomyopathy, a kind of cardiovascular dysfunction induced by severe sepsis and septic shock, is manifested by low cardiac output and is closely related to higher mortality in septic shock patients. International guidelines (2016) recommend a trial of dobutamine in the case of tissue hypoperfusion or myocardial dysfunction.^[13]^ Dobutamine can improve myocardial contractility in patients with septic shock by exciting the myocardial betareceptor.^[14]^ Although it has also been found that dobutamine can improve the microcirculation and peripheral tissue, while some clinical trials suggested that dobutamine cannot improve the outcome of septic shock patients, and even increase the mortality of 90days.^[7]^ Levosimendan, as a calcium sensitizer, is another attractive inotrope for cardiogenic shock in sepsis-induced cardiomyopathy.^[15]^ Unlike other inotropic agents, the positive inotropic effect of levosimendan is independent of the production of cyclic adenosine monophosphate, so it could minimize oxygen demand, arrhythmia, and catecholamines resistance. This property is of great significance for myocardial inhibition in septic patients under the hyperdynamic metabolic state. In addition, levosimendan could improve ATP-dependent potassium channels^[16]^: on the one hand, it could improve mitochondrial calcium overload, preserve high-energy phosphates, regulate the mitochondrial number, and exert relevant protective effects in ischemic myocardium; on the other hand, levosimendan can open potassium channels of smooth muscle and regulate intracellular Ca^2+^ concentration, resulting in vasodilation and decreased peripheral vascular resistance. This characteristic is of great significance for myocardial depression in septic shock with high dynamic metabolism.

This is the first meta-analysis to compare the efficacy and safety of levosimendan versus dobutamine for the treatment of sepsisinduced cardiomyopathy. More high-quality randomized controlled trials should be conducted to provide reasonable and firm evidence for patients.

## Author contributions

**Data curation:** Xianhuan Zhang.

**Investigation:** Yanan Zhu.

**Writing** - **original draft:** Jun Guo.

**Writing** - **review & editing:** Qiong Cheng.
